# Identification of Hub Genes and MicroRNAs Associated With Idiopathic Pulmonary Arterial Hypertension by Integrated Bioinformatics Analyses

**DOI:** 10.3389/fgene.2021.636934

**Published:** 2021-04-29

**Authors:** Xue Qiu, Jinyan Lin, Bixiao Liang, Yanbing Chen, Guoqun Liu, Jing Zheng

**Affiliations:** ^1^Department of Cardiology, The First Affiliated Hospital of Guangxi Medical University, Nanning, China; ^2^The First Clinical Medical School, Guangxi Medical University, Nanning, China

**Keywords:** idiopathic pulmonary arterial hypertension, differentially expressed genes, functional enrichment analysis, weighted gene co-expression network analysis, hub genes

## Abstract

**Objective:**

The aim of this study is the identification of hub genes associated with idiopathic pulmonary arterial hypertension (IPAH).

**Materials and Methods:**

GSE15197 gene expression data was downloaded from the Gene Expression Omnibus (GEO) database. Differentially expressed genes (DEGs) were identified by screening IPAH patients and controls. The 5,000 genes with the greatest variances were analyzed using a weighted gene co-expression network analysis (WGCNA). Modules with the strongest correlation with IPAH were chosen, followed by a functional enrichment analysis. Protein–protein interaction (PPI) networks were constructed to identify hub gene candidates using calculated degrees. Real hub genes were found from the overlap of DEGs and candidate hub genes. microRNAs (miRNAs) targeting real hub genes were found by screening miRNet 2.0. The most important IPAH miRNAs were identified.

**Results:**

There were 4,395 DEGs identified. WGCNA indicated that green and brown modules associated most strongly with IPAH. Functional enrichment analysis showed that green and brown module genes were mainly involved in protein digestion and absorption and proteoglycans in cancer, respectively. The top ten candidate hub genes in green and brown modules were identified, respectively. After overlapping with DEGs, 11 real hub genes were identified: *EP300*, *MMP2*, *CDH2*, *CDK2*, *GNG10*, *ALB*, *SMC2*, *DHX15*, *CUL3*, *BTBD1*, and *LTN1*. These genes were expressed with significant differences in IPAH versus controls, indicating a high diagnostic ability. The miRNA–gene network showed that hsa-mir-1-3p could associate with IPAH.

**Conclusion:**

*EP300*, *MMP2*, *CDH2*, *CDK2*, *GNG10*, *ALB*, *SMC2*, *DHX15*, *CUL3*, *BTBD1*, and *LTN1* may play essential roles in IPAH. Predicted miRNA hsa-mir-1-3p could regulate gene expression in IPAH. Such hub genes may contribute to the pathology and progression in IPAH, providing potential diagnostic and therapeutic opportunities for IPAH patients.

## Introduction

Idiopathic pulmonary arterial hypertension (IPAH) is a pulmonary proliferative vasculopathy (Gallo de Moraes et al., 2016). Pathological changes including plexiform lesions, cellular proliferation, fibrosis, *in situ* thrombosis of the small pulmonary arteries and arterioles, and angiogenic dysfunction, leading to increased pulmonary vascular resistance, result in IPAH (Barnes et al., 2019). The incidence of IPAH is approximately four to six per million globally. When left untreated, IPAH eventually leads to right heart failure and death (Pahal and Sharma, 2020). IPAH remains intractable, with a 51% 5-year survival rate (Barnes et al., 2019). In the last 20 years, new therapies have been developed, improving hemodynamics and long-term prognosis (Wang Y. et al., 2019). For those not sensitive to therapy, surgery such as atrial septostomy and lung transplantation are options, although the prognosis is poor (Pahal and Sharma, 2020).

Many genes and microRNAs (miRNAs) have been shown to be involved in IPAH. A meta-analysis suggested the serotonin transporter (*SERT*) is associated with IPAH risk, and those with long genotypes have greater incidence of IPAH than those with short genotypes (Zhang et al., 2013). Mutations in the human bone morphogenetic protein 9 (*BMP9*) gene reduced anti-apoptosis in pulmonary arterial endothelial cells (Wang X. J. et al., 2019). A greater expression of miR-199b-5p accelerated hemodynamics and pulmonary vascular remodeling (Wu et al., 2016). Silencing of miR23a increased the expression of PGC1α, leading to IPAH progression (Sarrion et al., 2015). However, the genetic mechanisms underlying IPAH pathology remain unclear. More studies are required to explore the pathogenesis, potential drug targets, and diagnostic biomarkers of IPAH.

A systems biology analysis of gene expression and regulation has become an effective method for exploring disease pathogenesis. A weighted gene co-expression network analysis (WGCNA) can identify correlations between genes and microarray samples (Wang T. et al., 2019). Clustering genes with similar expression profiles can identify the association of genes and clinical traits. Thus, WGCNA can be used to find hub genes associated with a specific disease, including cardiovascular disease (Zheng et al., 2020) and cancer (Hu et al., 2020; Shi et al., 2021).

**FIGURE 1:**
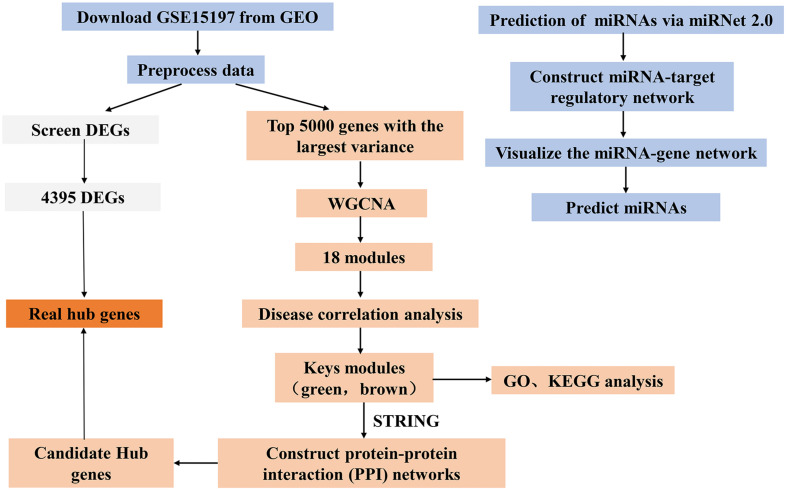
Flow chart for this study. miRNA, microRNA.

Although cardiovascular disease is well studied, there are few bioinformatics analyses of IPAH. Here, a co-expression network was constructed to identify genes related to IPAH pathogenesis, providing new routes to diagnose and treat IPAH. This study should provide novel biomarkers associated with IPAH pathogenesis and progression, which may be useful as potential therapeutic targets in IPAH. The flow diagram of the work is shown in Figure 1.

## Materials and Methods

### Data Sources

Microarray expression data (GSE15197) was downloaded from the Gene Expression Omnibus (GEO)^1^ database, representing a GPL6480 Agilent-014850 Whole Human Genome Microarray 4 × 44K G4112F (Probe Name version). GSE15197 includes 31 lung tissue specimens from 18 IPAH patients and 13 normal controls. Subject characteristics are presented in Supplementary Table 1. Platform information and probe annotation were extracted for additional analysis.

### Data Preprocessing and Differentially Expressed Genes Screening

The downloaded gene expression data (GSE15197.txt) was preprocessed using k-nearest neighbor (KNN) to supplement missing values. Expression levels were then normalized using a log2 transformation. When multiple probes map to the same gene, the average value was retained as the expression level. After pooling unmatched probes, 17,540 mRNAs were accessed. These samples were divided between IPAH and control groups. Differently expressed mRNAs (DEmRNAs) were screened using the limma package in R3.5.3 (Ritchie et al., 2015). A false discovery rate and the Benjamini–Hochberg method (Fu et al., 2014) were used to calculate fold changes (FC). | Log_2_FC| > 0.5 and adjusted *p*-value < 0.05 were used as thresholds.

### Weighted Gene Co-expression Network Analysis

To maintain sample diversity, the top 5,000 genes with the greatest variance between IPAH and normal controls were used to construct a co-expression network using the WGCNA package in R3.5.3 (Li et al., 2020; Tutorials for Wgcna R package, 2020; Zhang and Horvath, 2005). The “hclust” function was used for sample cluster analysis. Samples with heights over 115 were regarded as outliers. Other samples were used for calculating Pearson’s correlation coefficients. Scale independence and mean connectivity were calculated using the gradient method, with power values between 1 and 20. An appropriate soft threshold power of β was selected to meet the standard of a scale-free network (scale-free R2 ≥ 0.80) (Langfelder and Horvath, 2008) and ensure the network with enough information. The adjacency matrix was transformed into a topological overlap measure (TOM) matrix, which helped estimate the connectivity properties of the network (Iancu et al., 2015). Subsequently, the dynamic branch-cutting method was used to identify gene modules. Genes with similar gene expression profiles were placed in the same module. Each module contained genes with a minimum size of 30. The correlation between module eigengenes and clinical traits was analyzed and displayed as a heatmap. Modules that correlated most significantly with disease status were treated as key modules of IPAH. Gene significance (GS) represented the association between gene expression and each trait. Module membership (MM) was the correlation between gene expression and each module eigengene. The correlation between MM and GS was calculated using Pearson correlation analysis to validate module–trait associations (Zhang and Horvath, 2005).

### Functional Enrichment Analysis of Genes in the Key Module

Key modules showing the strongest correlations with IPAH were chosen for further analysis. The gene complement of each key module was examined using Gene Ontology (GO) and the Kyoto Encyclopedia of Genes and Genomes (KEGG) pathway enrichment analysis using the GEne SeT Analysis Toolkit^2^. The GO analysis annotates gene function at three levels: biological process (BP), cellular component (CC), and molecular function (MF). *P* < 0.05 was taken as statistically significant. The top 10 enriched items from each GO category and from KEGG were displayed using bubble charts.

### Identification of Protein–Protein Interaction Networks

To explore the potential roles of hub genes in the pathogenesis of IPAH, we used genes from all key modules to construct protein–protein interaction (PPI) networks using the STRING database^3^ (version 11.0). Cytoscape3.6.1 software was used for visualization and analysis (Shannon et al., 2003). Nodes represent proteins. Edges between nodes indicate the evidence of the supposed relationship between distinct nodes. The more and stronger connections a node has, the more likely it is to have an important role in IPAH pathogenesis. Cytoscape3.6.1 provides 11 methods for calculating the connections between nodes. Degree was chosen to represent connections between nodes. Genes with the top 10 degree in key modules were considered candidate hub genes.

### Identification of Real Hub Genes and Statistical Analysis

To increase the biological significance of candidate hub genes, we sought overlaps between candidates and differentially expressed genes (DEGs) to find real hub genes. Student’s *t*-tests were used to assess expression differences of 11 hub genes between IPAH and controls. Receiver operating characteristic (ROC) curves were used to evaluate the diagnostic ability of hub genes, with the area under the curve (AUC) representing sensitivity and specificity. All *P*-values were two sided. *P* < 0.05 was taken as statistically significant. Statistical analysis was performed using GraphPad Prism 8.0 (GraphPad Statistics Guide, 2020) and MedCalc 19.5.1 (Schoonjans et al., 1995).

### Construction of Potential miRNA-Target Regulatory Networks

We used miRNet 2.0^4^ to search for miRNAs targeting real hub genes and visualized the miRNA-target regulatory network (Fan et al., 2016). miRNAs with degrees 3 or above are shown.

## Results

### DEGs Screening

There were 4,395 differently expressed genes between IPAH and controls. This included 2,529 upregulated and 1,866 downregulated genes. The top 20 DEGs are listed in Supplementary Table 2.

### Construction and Analysis of Co-expression Network

**FIGURE 2:**
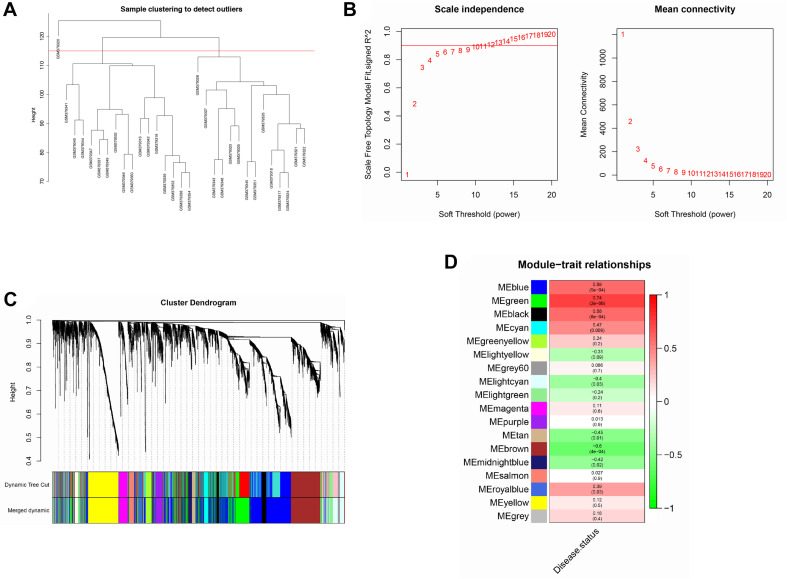
Construction of co-expression network. **(A)** Sample clustering analysis based on GSE15197. **(B)** Analysis of network topology for various soft-thresholding powers. **(C)** Clustering dendrogram of genes. Genes with similar expression patterns divided into same module. Each line of hierarchical clustering represents one gene. **(D)** Module–trait associations. Each cell contains the corresponding correlation and *P-*value.

**FIGURE 3:**
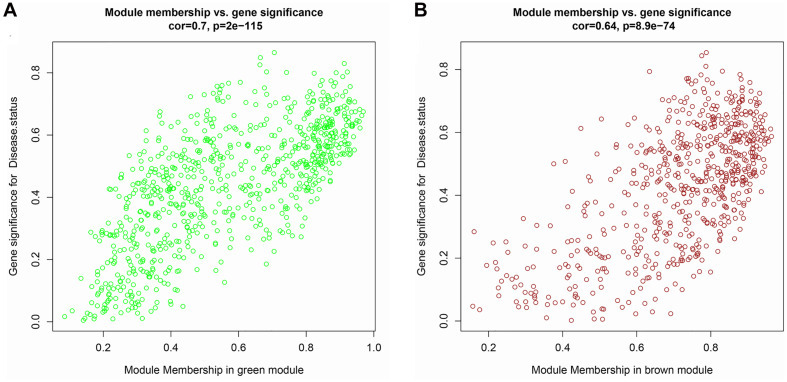
Scatter plot of module membership (MM) vs. gene significance (GS) in **(A)** green and **(B)** brown modules. MM presents the correlation between gene expression and each module eigengene. GS represents the association between gene expression and each trait. In both modules, GS and MM have a high correlation.

The top 5,000 genes were selected for construction of a co-expression network. Results of the cluster analysis are shown in Figure 2A. After clustering, GSM379320 was identified as an outlier, so has been excluded. The other 30 samples were used to construct a WGCNA network. To ensure a scale-free network and greater mean connectivity, a power of β = 6 was chosen (Figure 2B). As shown in Figure 2C, 18 gene modules were identified using the dynamic branch-cutting approach, with a merge cut height of 0.25. Genes in the same module had similar expression profiles. From the module–trait relationships analysis (Figure 2D), we found that the green (*r* = 0.74, *p* < 0.001), blue (*r* = 0.59, *p* < 0.001), black (*r* = 0.58, *p* < 0.001), and brown modules (*r* = −0.60, *p* < 0.001) were strongly related to the IPAH disease status. The green module had the strongest positive relation with IPAH, and the brown module had the strongest negative relation. We considered these two modules as the key IPAH modules, selecting them for further analysis. Associations between MM and GS for disease status were calculated. Significant correlations between MM and GS for IPAH in the green and brown modules are presented in Figures 3A,B.

### Functional Enrichment Analysis of Genes in the Green and Brown Modules

**FIGURE 4:**
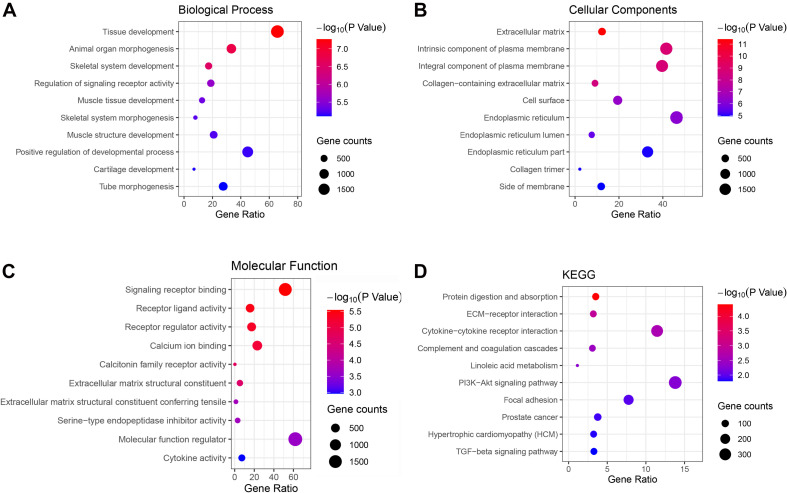
Gene Ontology (GO) terms in **(A)** biological process, **(B)** cellular component, **(C)** molecular function, and **(D)** the Kyoto Encyclopedia of Genes and Genomes (KEGG) pathway of genes in the green module.

**FIGURE 5:**
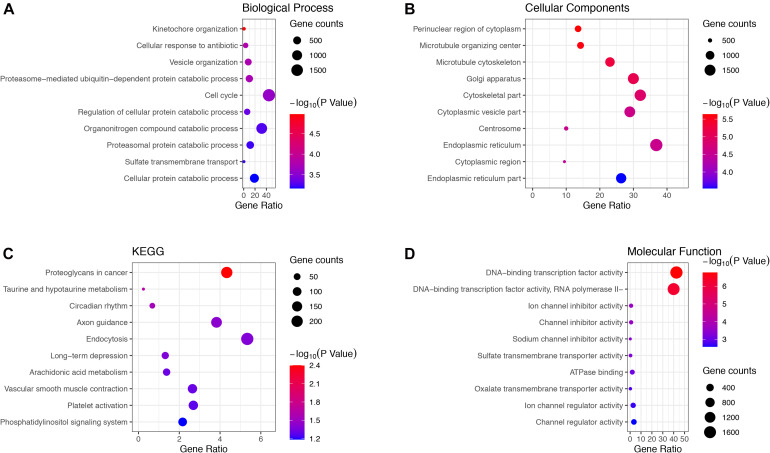
Gene Ontology terms in **(A)** biological process, **(B)** cellular component, **(C)** molecular function and, **(D)** KEGG pathway of genes in the brown module.

Genes in the green and brown modules were analyzed using GO and KEGG enrichment analysis. As shown in Figure 4, the results of GO analysis indicated that the green module genes were primarily associated with tissue development, extracellular matrix, and signaling receptor binding. KEGG analysis suggested that the green module genes were mainly enriched with protein digestion and absorption. GO enrichment results indicated that the brown module genes were significantly associated with kinetochore organization, the perinuclear region of the cytoplasm, and DNA-binding transcription factor activity. Moreover, KEGG analysis suggested that the brown module genes were mainly enriched in proteoglycans in cancer (Figure 5).

### PPI Network Analysis of Genes in the Green and Brown Modules

**FIGURE 6:**
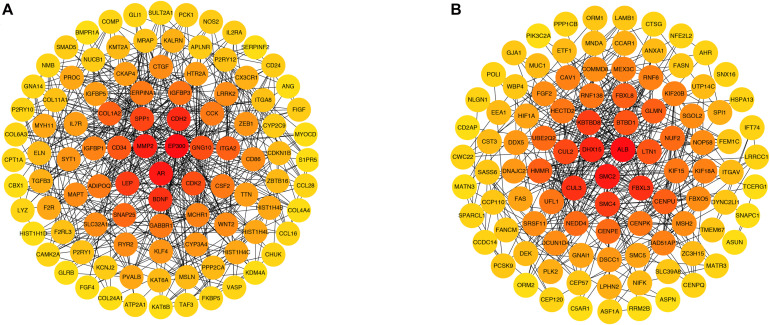
Protein–protein interaction networks of genes in **(A)** green and **(B)** brown modules. Each node represents a gene, and each node is connected by a degree. The color changes from red to yellow as one moves from the inside toward the outside, and the degree decreases from red to yellow. The genes with top 100 degrees and genes with the same degrees as the 100th gene were also presented.

Green and brown module genes were used to construct a PPI network using the STRING database. The combined score was > 0.4. The top 100 genes from the green and brown modules as ranked by degree were visualized using Cytoscape3.6.1 (Figure 6). The 10 genes with the highest degree from each module were considered candidate IPAH hub genes.

In the green module, candidate hub genes were as follows: E1A binding protein p300 (*EP300*, degree 51); androgen receptor (*AR*, degree 39); matrix metallopeptidase 2 (*MMP2*, degree 36); cadherin 2 (*CDH2*, degree 35); brain-derived neurotrophic factor (*BDNF*, degree 33); leptin (*LEP*, degree 32); secreted phosphoprotein 1 (*SPP1*, degree 30); cyclin-dependent kinase 2 (*CDK2*, degree 27); G protein subunit gamma 10 (*GNG10*, degree 27); and CD34 (*CD34*, degree 26).

In the brown module, the candidate hub genes included the following: albumin (*ALB*, degree 37), structural maintenance of chromosomes 2 (*SMC2*, degree 24); DEAH-box helicase 15 (*DHX15*, degree 22); cullin 3 (*CUL3*, degree 21); F-box and leucine rich repeat protein 3 (*FBXL3*, degree 21); kelch repeat and BTB domain containing 8 (*KBTBD8*, degree 20); structural maintenance of chromosomes 4 (*SMC4*, degree 20); cullin 2 (*CUL2*, degree 19); BTB domain containing 1 (*BTBD1*, degree 18); and listerin E3 ubiquitin protein ligase 1 (*LTN1*,degree 18).

### Identification and Verification of Real Hub Gene Expression and ROC Curve Analysis

**FIGURE 7:**
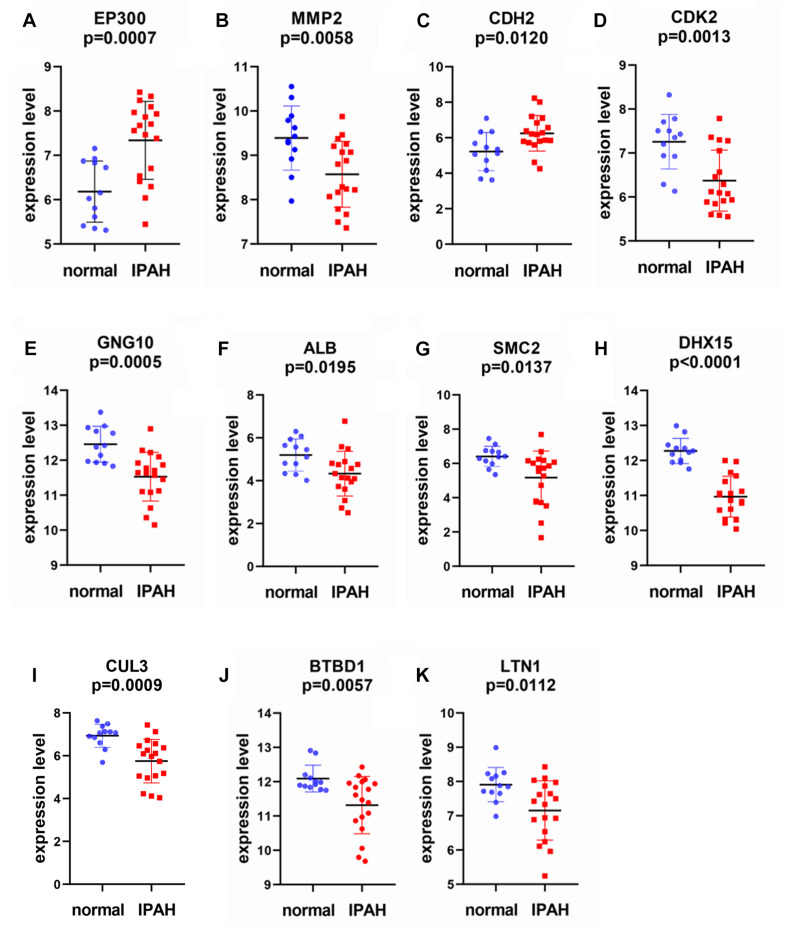
Scatter diagrams for the expressions of real hub genes in GSE15197. **(A)** *EP300*, **(B)** *MMP2*, **(C)** *CDH2*, **(D)** *CDK2*, **(E)** *GNG10*, **(F)** *ALB*, **(G)** *SMC2*, **(H)** *DHX15*, **(I)** *CUL3*, **(J)** *BTBD1*, and **(K)** *LTN1*. Each point represents a sample. In GSE15197, there are 18 IPAH samples and 12 normal samples. *EP300*, E1A binding protein p300; *MMP2*, matrix metallopeptidase 2; *CDH2*, cadherin 2; *CDK2*, cyclin-dependent kinase 2; *GNG10*, G protein subunit gamma 10; *ALB*, albumin; *SMC2*, structural maintenance of chromosomes 2; *DHX15*, DEAH-box helicase 15; *CUL3*, cullin 3; *BTBD1*, BTB domain containing 1; *LTN1*, listerin E3 ubiquitin protein ligase 1.

**FIGURE 8:**
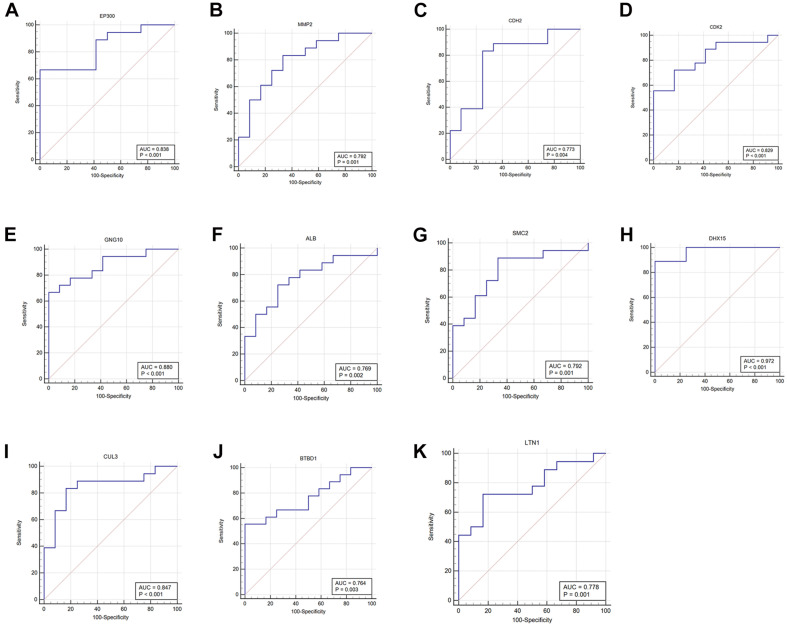
ROC curves of the hub genes. **(A)** *EP300*, **(B)** *MMP2*, **(C)** *CDH2*, **(D)** *CDK2*, **(E)** *GNG10*, **(F)** *ALB*, **(G)** *SMC2*, **(H)** *DHX15*, **(I)** *CUL3*, **(J)** *BTBD1*, and **(K)** *LTN1*. ROC, receiver operating characteristic; *EP300*, E1A binding protein p300; *MMP2*, matrix metallopeptidase 2; *CDH2*, cadherin 2; *CDK2*, cyclin-dependent kinase 2; *GNG10*, G protein subunit gamma 10; *ALB*, albumin; *SMC2*, structural maintenance of chromosomes 2; *DHX15*, DEAH-box helicase 15; *CUL3*, cullin 3; *BTBD1*, BTB domain containing 1; *LTN1*, listerin E3 ubiquitin protein ligase 1.

After overlapping with DEGs, we selected 11 real hub genes: *EP300*, *MMP2*, *CDH2*, *CDK2*, *GNG10*, *ALB*, *SMC2*, *DHX15*, *CUL3*, *BTBD1*, and *LTN1* (see Supplementary Table 3). As shown in Figure 7, the expression level differences in real hub genes were statistically significant. *EP300* and *CDH2* were highly expressed in IPAH patients. Other hub genes showed a greater expression in controls. ROC curve analysis indicated that 11 real hub genes discriminated strongly between IPAH patients and controls (Figure 8). Real hub genes might have important roles in IPAH pathogenesis and development.

### Prediction of Potential miRNA-Target Regulatory Networks

**FIGURE 9:**
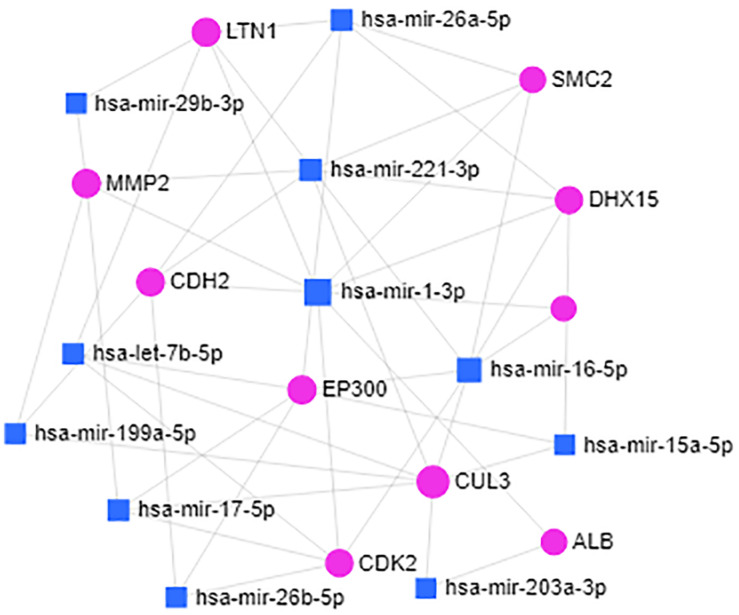
miRNA–target regulatory network. The blue square represents miRNAs targeting the real hub genes, and the red circle represents the real hub genes. The bigger the circle or square, the higher the degree.

We searched for miRNAs targeting real hub genes using the miRNet 2.0 database. The resulting miRNA-target network was visualized using miRNet 2.0 (Figure 9). Only miRNAs with a degree of 3 or over were displayed. We found that hsa-mir-1-3p had the highest degree (degree 8). We speculated that hsa-mir-1-3p could be the most crucial miRNA in IPAH pathogenesis and development.

## Discussion

IPAH is a rare but severe cardiopulmonary disease associated with progressive deterioration. There are many molecular mechanisms underlying IPAH, and extant treatments for IPAH are limited (Yanai et al., 2017). To find new targets or therapies, it is essential to explore IPAH hub genes. Bioinformatics analysis of microarray data has been used widely to identify disease-associated hub genes (Yin et al., 2018).

In this study, DEGs from GSE15197 were analyzed. There were 4,395 significantly differentially expressed genes identified in IPAH and normal lung tissue. These DEGs may play important biological roles. We undertook WGCNA on GSE15197, identifying the green and brown modules as key modules. We also conducted GO and KEGG analyses of these modules. The GO analysis showed that the green and brown module genes were enriched, respectively, in tissue development and kinetochore organization. The KEGG analysis indicated that the green and brown modules genes were involved, respectively, in protein digestion and absorption and proteoglycans in cancer. By overlapping DEGs and candidate hub genes obtained from WGCNA, we found 11 real hub genes associated with IPAH: *EP300*, *MMP2*, *CDH2*, *CDK2*, *GNG10*, *ALB*, *SMC2*, *DHX15*, *CUL3*, *BTBD1*, and *LTN1*.

Among the 11 hub genes, *EP300* and *CDH2* are upregulated hub genes. *EP300* encodes histone acetyltransferase, which regulates gene transcription by binding chromatin in the cell nucleus (Sen et al., 2019). It has been linked to arterial stiffness prior to hypertension, increase of pulse pressure, and structural vessel wall changes (Herrera et al., 2014). *EP300* can function as a regulatory factor during vascular endothelial growth factor A-induced angiogenesis (Sacilotto et al., 2016). The overexpression of *EP300* increases lung fibrotic hallmarks in a bleomycin mouse model (Rubio et al., 2019). Cardiac miR-133a overexpression in diabetes inhibits *EP300*, preventing early cardiac fibrosis (Chen et al., 2014). We reason that *EP300* may promote IPAH development. Although much evidence supports a role for *EP300* in vascular disease, its mechanism in IPAH remains unclear. Further studies are required to explore how *EP300* participates in IPAH progression.

*CDH2* encodes cadherin 2 or N-cadherin (Mayosi et al., 2017). Significant *CDH2* expression promotes endothelial cell proliferation and vascular smooth muscle cell migration (Lyon et al., 2010; Zhuo et al., 2019), causing intimal thickening, and could drive vascular remodeling in IPAH. Moreover, *CDH2* mutations are associated with arrhythmogenic cardiomyopathy (Mayosi et al., 2017; Ghidoni et al., 2021).

*MMP2* is a proteolytic enzyme that contributes to vascular protein degradation and aortic wall destruction (Longo et al., 2002; Li et al., 2019). Much evidence suggest that *MMP2* is associated with an increased risk of cardiovascular diseases, such as myocardial infarction (Alp et al., 2011) and degenerative mitral valve disease (Balistreri et al., 2016). Rat vascular smooth muscle cell migration can be inhibited by the *c-Myc/MMP2* and *ROCK/JNK* signaling pathways (Luo et al., 2019). Higher ratios of *MMP2/TIMP4* in plasma predict a significantly higher risk of death or clinical deterioration in IPAH patients (Wetzl et al., 2017). Activation of *MMP2* is increased in smooth muscle cells of IPAH patients, contributing to smooth muscle cell migration and proliferation (Lepetit et al., 2005).

*CDK2* encodes a form of protein-dependent kinases (Nathans et al., 2021), involved in cell cycle regulation, with a critical role during the G1 to S phase transition. Decreased *CDK2* expression can block cell cycle progression and inhibit the proliferation of pulmonary artery smooth muscle cells. Proliferative PASMCs exist in PAH patients and are closely related to vascular remodeling (Yue et al., 2020).

*ALB* encodes albumin, the most abundant protein in human blood, which plays a key role in regulating blood plasma colloid osmotic pressure. In IPAH, pulmonary vascular pressure is considerably elevated, leading to endothelial dysfunction and capillary leakage easily. In turn, this leads to loss of plasma proteins including serum albumin levels. Lower albumin levels may reflect disease progression and predict worse survival rates (Snipelisky et al., 2018).

*CUL3* encodes cullin 3, a cullin protein family member, critical to maintaining the integrity of the endothelial barrier (Kovačević et al., 2018). Decreased expression of *CUL3* may occur in IPAH, resulting in the destruction of blood vessel architecture, cell proliferation, and vascular remodeling (Sakaue et al., 2017). Rats with *CUL3* mutations exhibit arterial hypertension, while patients with *CUL3* mutations present severe early-onset hypertension, vascular dysfunction, and arterial stiffness due to deficiency of vascular smooth muscle (Abdel Khalek et al., 2019).

*GNG10* encodes a subtype of the G-protein γ subunit involved in suppressing heart rate (Senarath et al., 2018) and regulating the cell cycle (Clapham and Neer, 1997). The protein encoded by *SMC2* belongs to the condensin complex, which maintains chromosome stability (Feng et al., 2019). *DHX15*, a member of the DEAH-box RNA helicase family, participates in modulating pre-mRNA splicing (Jing et al., 2018). *BTBD1* encodes a 482-amino-acid protein involved in protein–protein interactions. The expression of *BTBD1* in the heart is enhanced (Carim-Todd et al., 2001; Xu et al., 2002). *LTN1* encodes listerin E3 ubiquitin protein ligase 1, which functions as an E3 ubiquitin ligase (Chu et al., 2009). However, there is no research indicating a relationship between these five genes and IPAH. We propose that these genes are novel genes related to IPAH, suggesting the need for future study.

miRNAs represent novel potential therapeutic targets for many diseases including heart failure (Akat et al., 2014), acute myocardial infarction (Gupta et al., 2016), arrhythmias (den Hoed et al., 2013), and pulmonary hypertension (Zhou et al., 2018). We created a miRNA-target network to explore potential miRNAs in IPAH. The network suggested that hsa-mir-1-3p might regulate gene expression in IPAH. Previous studies have shown elevated hsa-mir-1-3p in perioperative myocardial injury patients (May et al., 2020). Further research is needed to verify the mechanism linking hsa-mir-1-3p to IPAH.

We also examined expression levels in IPAH versus controls and the diagnostic power of the 11 identified hub genes using the ROC curve analysis. The expression of the 11 hub genes showed significant differences between IPAH and controls. A previous work has identified diagnostic IPAH biomarkers in plasma (Wang T. et al., 2019). Here, we used lung microarray data, not plasma, to identify hub genes using WGCNA. We consider that this result is more reliable for spatial specificity of gene expression. The 11 genes may all have a considerable diagnostic ability and be better candidate targets, although lung tissues cannot be obtained as easily as plasma samples.

The principal limitation of our study is the lack of independent experimental validation. Although 11 hub genes were identified, their involvement in IPAH pathogenesis has not been experimentally validated using *in vivo* or *in vitro* experimentation, and their possible mechanisms of action remain unclear. Further research is needed to validate our results and to explore the specific mechanisms of action for each gene.

## Conclusion

In conclusion, our results indicate that the 11 real hub genes may play critical roles in IPAH. *EP300* and *CDH2* are the upregulated hub genes, while *MMP2*, *CDK2*, *GNG10*, *ALB*, *SMC2*, *DHX15*, *CUL3*, *BTBD1*, and *LTN1* are the downregulated hub genes. Predicted miRNA hsa-mir-1-3p might regulate gene expression in IPAH. These hub genes could contribute to the pathology and progression of IPAH and may be candidate targets for IPAH treatment.

## Data Availability Statement

Publicly available datasets were analyzed in this study. This data can be found here: GSE15197.

## Author Contributions

XQ, JL, and JZ designed the research, analyzed the data, and wrote the manuscript. BL, YC, and GL participated in data preparation, analysis of data, and figures preparation. JZ revised the manuscript. All authors read and approved the manuscript for publication.

## Conflict of Interest

The authors declare that the research was conducted in the absence of any commercial or financial relationships that could be construed as a potential conflict of interest.

## Footnotes

1 https://www.ncbi.nlm.nih.gov/geo/

2 http://www.webgestalt.org/

3 https://string-db.org

4 https://www.mirnet.ca/
