# Supplementary material for: Identification of Hub Genes and MicroRNAs Associated With Idiopathic Pulmonary Arterial Hypertension by Integrated Bioinformatics Analyses
Source: Front Genet. 2021 Apr 29;12:667406. doi: 10.3389/fgene.2021.636934 (PMC8117102; doi:10.3389/fgene.2021.636934)
Supplement: Supplementary file 1 [file Table_1.docx]

**Supplement** **Table 1** Subject characteristics

| **Cohort** | **n** | **Age,yr** | **Male/female,N** | **Race** | **PVRI,Wood units** | **MPVP,mmHg** |
| --- | --- | --- | --- | --- | --- | --- |
| IPAH | 18 | 44±10 | 7/11 | White 17;Native American 1 | 20±9 | 55±7 |
| Normal controls | 13 | 60±11 | 5/8 | White 12;Native American 1 |  |  |
| IPAH, idiopathic pulmonary arterial hypertension; PVRI, pulmonary vascular resistance index; MPAP, mean pulmonary arterial pressure. | | | | | | |
| Data are presented as mean± SD or number (%).  Cited from PMID: 20081107; DOI: 10.1152/ajpheart.00254.2009. | | | | | |  |
