# Supplementary material for: Identification of Hub Genes and MicroRNAs Associated With Idiopathic Pulmonary Arterial Hypertension by Integrated Bioinformatics Analyses
Source: Front Genet. 2021 Apr 29;12:667406. doi: 10.3389/fgene.2021.636934 (PMC8117102; doi:10.3389/fgene.2021.636934)
Supplement: Supplementary file 2 [file Table_2.docx]

**Supplement** **Table 2** Top twenty up and down regulated genes

| **Upregulated genes** | | |  | **Downregulated genes** | | |
| --- | --- | --- | --- | --- | --- | --- |
| **Gene symbol** | **adjust p.value** | **Log_2_FC** |  | **Gene symbol** | **adjust p.value** | **Log_2_FC** |
| ARHGEF35 | 0.000313048 | 3.384928 |  | CHL1 | 9.67E-09 | -2.67545 |
| CABP7 | 0.005466725 | 3.336347 |  | IL13 | 0.001558 | -2.66807 |
| POU3F3 | 0.006892997 | 3.162362 |  | TCTEX1D1 | 0.000715 | -2.34293 |
| GSTT1 | 0.01003059 | 3.12468 |  | DKK1 | 0.000209 | -2.29332 |
| GPR78 | 0.019294188 | 3.121513 |  | MIR100HG | 0.000116 | -2.20788 |
| NEUROG3 | 0.002940651 | 2.784425 |  | IL33 | 0.000209 | -2.08702 |
| ARL17B | 0.000191644 | 2.766663 |  | PROK2 | 0.01607 | -2.08615 |
| IFITM5 | 0.00953795 | 2.735882 |  | RNASE2 | 0.003429 | -2.07872 |
| SOST | 0.007832613 | 2.695217 |  | KLRF1 | 0.003892 | -2.07069 |
| FLJ22184 | 0.012243699 | 2.678786 |  | TM4SF1 | 5.47E-05 | -2.01305 |
| RNU12 | 0.000208505 | 2.670408 |  | CCL7 | 0.017871 | -2.00927 |
| PF4 | 0.001963927 | 2.637637 |  | IL6 | 0.016022 | -2.00084 |
| COX6A2 | 0.014306087 | 2.631907 |  | POM121L9P | 0.000212 | -1.94029 |
| NEUROG1 | 0.012896143 | 2.619405 |  | CYP4Z1 | 0.00075 | -1.90846 |
| HIST1H4E | 0.001227538 | 2.597885 |  | TUBE1 | 3.78E-05 | -1.89134 |
| GPR153 | 0.0191604 | 2.55048 |  | RGS13 | 0.00633 | -1.87297 |
| C9orf62 | 0.010603107 | 2.501465 |  | VIP | 0.000754 | -1.86558 |
| HOXA6 | 7.43E-06 | 2.461552 |  | SNORD22 | 0.000122 | -1.86254 |
| PCSK1N | 0.003360936 | 2.449584 |  | CX3CR1 | 0.007652 | -1.85931 |
| LOC145694 | 0.008572455 | 2.442861 |  | C3orf64 | 0.001512 | -1.85348 |
| FC, fold change. | | | | | | |
