# Supplementary material for: Identification of Hub Genes and MicroRNAs Associated With Idiopathic Pulmonary Arterial Hypertension by Integrated Bioinformatics Analyses
Source: Front Genet. 2021 Apr 29;12:667406. doi: 10.3389/fgene.2021.636934 (PMC8117102; doi:10.3389/fgene.2021.636934)
Supplement: Supplementary file 3 [file Table_3.docx]

**Supplement Table 3** Real hub genes identified in the PPI network for the green and brown modules

| **Module** | **Gene Symbol** | **Description** | **Degree** | **log_2_FC** | **adjust P value** |
| --- | --- | --- | --- | --- | --- |
| Green | *EP300* | E1A binding protein p300 | 51 | 1.11 | 0.0041 |
|  | *MMP2* | Matrix metallopeptidase 2 | 36 | -0.88 | 0.0112 |
|  | *CDH2* | cadherin 2 | 35 | 0.94 | 0.0462 |
|  | *CDK2* | cyclin dependent kinase 2 | 27 | -0.81 | 0.0109 |
|  | *GNG10* | G protein subunit gamma 10 | 27 | -0.91 | 0.0028 |
| Brown | *ALB* | Albumin | 37 | -0.93 | 0.0300 |
|  | *SMC2* | Structural maintenance of chromosomes 2 | 24 | -1.24 | 0.0284 |
|  | *DHX15* | DEAH-box helicase 15 | 22 | -1.32 | 0.0000 |
|  | *CUL3* | Cullin 3 | 21 | -1.20 | 0.0033 |
|  | *BTBD1* | BTB domain containing 1 | 18 | -0.77 | 0.0162 |
|  | *LTN1* | Listerin E3 ubiquitin protein ligase 1 | 28 | -0.73 | 0.0308 |
| PPI, protein-protein interaction; FC, fold change. | | | | | |
