# Supplementary material for: Identification of Hub Genes and MicroRNAs Associated With Idiopathic Pulmonary Arterial Hypertension by Integrated Bioinformatics Analyses
Source: Front Genet. 2021 Apr 29;12:667406. doi: 10.3389/fgene.2021.636934 (PMC8117102; doi:10.3389/fgene.2021.636934)
Supplement: Supplementary file 4 [file Data_Sheet_1.docx]

The code used for DEGs screening:

logFoldChange=0.5

adjustP=0.05

install.packages("limma")

library(limma)

setwd(""C:\\Users\\M\\Desktop\\work"") rt=read.table("normalize.txt",sep="\t",header=T,check.names=F,quote = "")

rt=as.matrix(rt)

rownames(rt)=rt[,1]

exp=rt[,2:ncol(rt)]

dimnames=list(rownames(exp),colnames(exp))

rt=matrix(as.numeric(as.matrix(exp)),nrow=nrow(exp),dimnames=dimnames)

rt=avereps(rt)

rt=log2(rt+1)

type=c(rep("normal",13),rep("IPAH",18))

design <- model.matrix(~0+factor(type))

colnames(design) <- c("normal","IPAH")

fit <- lmFit(rt,design)

cont.matrix<-makeContrasts(treat-con,levels=design)

fit2 <- contrasts.fit(fit, cont.matrix)

fit2 <- eBayes(fit2)

allDiff=topTable(fit2,adjust='fdr',number=200000)

allOut=rbind(id=colnames(allDiff),allDiff)

write.table(allOut,file="limmaTab.xls",sep="\t",quote=F,col.names=F)

diffSig <- allDiff[with(allDiff, (abs(logFC)>logFoldChange & adj.P.Val < adjustP )), ]

diffOut=rbind(id=colnames(diffSig),diffSig)

write.table(diffOut,file="diff.xls",sep="\t",quote=F,col.names=F)

hmExp=rt[rownames(diffSig),]

diffExp=rbind(id=colnames(hmExp),hmExp)

write.table(diffExp,file="diffExp.txt",sep="\t",quote=F,col.names=F)

dev.off()

The code used for WGCNA network:

install.packages ("GO.db", "preprocessCore", "impute")

install.packages(c("matrixStats", "Hmisc", "splines", "foreach", "doParallel", "reshape", "fastcluster", "dynamicTreeCut", "survival"))

install.packages("WGCNA")

setwd("C:\\Users\\M\\Desktop\\work")

library("WGCNA")

rt=read.table("input.txt",sep="\t",header=T,check.names=F)

dim(rt)

datExpr0 = as.data.frame(t(rt[,-1]))

names(datExpr0) = rt[,1]

rownames(datExpr0) = names(rt[,-1])

gsg = goodSamplesGenes(datExpr0, verbose = 3)

gsg$allOK

if (!gsg$allOK)

{

if (sum(!gsg$goodGenes)>0)

printFlush(paste("Removing genes:", paste(names(datExpr0)[!gsg$goodGenes], collapse = ", ")))

if (sum(!gsg$goodSamples)>0)

printFlush(paste("Removing samples:", paste(rownames(datExpr0)[!gsg$goodSamples], collapse = ", ")))

datExpr0 = datExpr0[gsg$goodSamples, gsg$goodGenes]

}

meanFPKM=0.5

n=nrow(datExpr0)

datExpr0[n+1,]=apply(datExpr0[c(1:nrow(datExpr0)),],2,mean)

datExpr0=datExpr0[1:n,datExpr0[n+1,] > meanFPKM]

filtered_fpkm=t(datExpr0)

filtered_fpkm=data.frame(rownames(filtered_fpkm),filtered_fpkm)

names(filtered_fpkm)[1]="sample"

head(filtered_fpkm)

sampleTree = hclust(dist(datExpr0), method = "average")

pdf(file = "1_sample_cluster.pdf", width = 12, height = 9)

par(cex = 0.6)

par(mar = c(0,4,2,0))

plot(sampleTree, main = "Sample clustering to detect outliers", sub="", xlab="", cex.lab = 1.5,

cex.axis = 1.5, cex.main = 2)

abline(h = 115, col = "red")

dev.off()

traitData = read.table("type.txt",row.names=1,header=T,comment.char = "",check.names=F)

dim(traitData)

names(traitData)

fpkmSamples = rownames(datExpr0)

traitSamples =rownames(traitData)

traitRows = match(fpkmSamples, traitSamples)

datTraits = traitData[traitRows,]

rownames(datTraits)

collectGarbage()

sampleTree2 = hclust(dist(datExpr0), method = "average")

traitColors = numbers2colors(datTraits, signed = FALSE)

pdf(file="2_sample_heatmap.pdf",width=12,height=12)

plotDendroAndColors(sampleTree2, traitColors,

groupLabels = names(datTraits),

main = "Sample dendrogram and trait heatmap")

dev.off()

enableWGCNAThreads()

powers = c(1:20)

sft = pickSoftThreshold(datExpr0, powerVector = powers, verbose = 5)

pdf(file="3_scale_independence.pdf",width=9,height=5)

par(mfrow = c(1,2))

cex1 = 0.9

plot(sft$fitIndices[,1], -sign(sft$fitIndices[,3])*sft$fitIndices[,2],

xlab="Soft Threshold (power)",ylab="Scale Free Topology Model Fit,signed R^2",type="n",

main = paste("Scale independence"));

text(sft$fitIndices[,1], -sign(sft$fitIndices[,3])*sft$fitIndices[,2],

labels=powers,cex=cex1,col="red");

abline(h=0.90,col="red")

plot(sft$fitIndices[,1], sft$fitIndices[,5],

xlab="Soft Threshold (power)",ylab="Mean Connectivity", type="n",

main = paste("Mean connectivity"))

text(sft$fitIndices[,1], sft$fitIndices[,5], labels=powers, cex=cex1,col="red")

dev.off()

sft

softPower =sft$powerEstimate

adjacency = adjacency(datExpr0, power = softPower)

TOM = TOMsimilarity(adjacency);

dissTOM = 1-TOM

geneTree = hclust(as.dist(dissTOM), method = "average");

pdf(file="4_gene_clustering.pdf",width=12,height=9)

plot(geneTree, xlab="", sub="", main = "Gene clustering on TOM-based dissimilarity",

labels = FALSE, hang = 0.04)

dev.off()

minModuleSize = 30

dynamicMods = cutreeDynamic(dendro = geneTree, distM = dissTOM,

deepSplit = 2, pamRespectsDendro = FALSE,

minClusterSize = minModuleSize);

table(dynamicMods)

dynamicColors = labels2colors(dynamicMods)

table(dynamicColors)

pdf(file="5_Dynamic_Tree.pdf",width=8,height=6)

plotDendroAndColors(geneTree, dynamicColors, "Dynamic Tree Cut",

dendroLabels = FALSE, hang = 0.03,

addGuide = TRUE, guideHang = 0.05,

main = "Gene dendrogram and module colors")

dev.off()

MEList = moduleEigengenes(datExpr0, colors = dynamicColors)

MEs = MEList$eigengenes

MEDiss = 1-cor(MEs);

METree = hclust(as.dist(MEDiss), method = "average")

pdf(file="6_Clustering_module.pdf",width=7,height=6)

plot(METree, main = "Clustering of module eigengenes",

xlab = "", sub = "")

MEDissThres = 0.25

abline(h=MEDissThres, col = "red")

dev.off()

merge = mergeCloseModules(datExpr0, dynamicColors, cutHeight = MEDissThres, verbose = 3)

mergedColors = merge$colors

mergedMEs = merge$newMEs

pdf(file="7_merged_dynamic.pdf", width = 9, height = 6)

plotDendroAndColors(geneTree, cbind(dynamicColors, mergedColors),

c("Dynamic Tree Cut", "Merged dynamic"),

dendroLabels = FALSE, hang = 0.03,

addGuide = TRUE, guideHang = 0.05)

dev.off()

moduleColors = mergedColors

table(moduleColors)

colorOrder = c("grey", standardColors(50))

moduleLabels = match(moduleColors, colorOrder)-1

MEs = mergedMEs

nGenes = ncol(datExpr0)

nSamples = nrow(datExpr0)

moduleTraitCor = cor(MEs, datTraits, use = "p")

moduleTraitPvalue = corPvalueStudent(moduleTraitCor, nSamples)

pdf(file="8_Module_trait.pdf",width=10,height=6)

textMatrix = paste(signif(moduleTraitCor, 2), "\n(",

signif(moduleTraitPvalue, 1), ")", sep = "")

dim(textMatrix) = dim(moduleTraitCor)

par(mar = c(5, 10, 3, 3))

labeledHeatmap(Matrix = moduleTraitCor,

xLabels = names(datTraits),

yLabels = names(MEs),

ySymbols = names(MEs),

colorLabels = FALSE,

colors = greenWhiteRed(50),

textMatrix = textMatrix,

setStdMargins = FALSE,

cex.text = 0.5,

zlim = c(-1,1),

main = paste("Module-trait relationships"))

dev.off()

modNames = substring(names(MEs), 3)

geneModuleMembership = as.data.frame(cor(datExpr0, MEs, use = "p"))

MMPvalue = as.data.frame(corPvalueStudent(as.matrix(geneModuleMembership), nSamples))

names(geneModuleMembership) = paste("MM", modNames, sep="")

names(MMPvalue) = paste("p.MM", modNames, sep="")

traitNames=names(datTraits)

geneTraitSignificance = as.data.frame(cor(datExpr0, datTraits, use = "p"))

GSPvalue = as.data.frame(corPvalueStudent(as.matrix(geneTraitSignificance), nSamples))

names(geneTraitSignificance) = paste("GS.", traitNames, sep="")

names(GSPvalue) = paste("p.GS.", traitNames, sep="")

picDir="module_trait"

dir.create(picDir)

for (trait in traitNames){

traitColumn=match(trait,traitNames)

for (module in modNames){

column = match(module, modNames)

moduleGenes = moduleColors==module

if (nrow(geneModuleMembership[moduleGenes,]) > 1){

pdfFile=paste("9_", trait, "_", module,".pdf",sep="")

outPdf=paste(picDir,pdfFile,sep="\\")

pdf(file=outPdf,width=7,height=7)

par(mfrow = c(1,1))

verboseScatterplot(abs(geneModuleMembership[moduleGenes, column]),

abs(geneTraitSignificance[moduleGenes, traitColumn]),

xlab = paste("Module Membership in", module, "module"),

ylab = paste("Gene significance for ",trait),

main = paste("Module membership vs. gene significance\n"),

cex.main = 1.2, cex.lab = 1.2, cex.axis = 1.2, col = module)

dev.off()

}

}

}

names(datExpr0)

probes = names(datExpr0)

geneInfo0 = data.frame(probes= probes,

moduleColor = moduleColors)

for (Tra in 1:ncol(geneTraitSignificance))

{

oldNames = names(geneInfo0)

geneInfo0 = data.frame(geneInfo0, geneTraitSignificance[,Tra],

GSPvalue[, Tra])

names(geneInfo0) = c(oldNames,names(geneTraitSignificance)[Tra],

names(GSPvalue)[Tra])

}

for (mod in 1:ncol(geneModuleMembership))

{

oldNames = names(geneInfo0)

geneInfo0 = data.frame(geneInfo0, geneModuleMembership[,mod],

MMPvalue[, mod])

names(geneInfo0) = c(oldNames,names(geneModuleMembership)[mod],

names(MMPvalue)[mod])

}

geneOrder =order(geneInfo0$moduleColor)

geneInfo = geneInfo0[geneOrder, ]

write.table(geneInfo, file = "GS_MM.xls",sep="\t",row.names=F)

nGenes = ncol(datExpr0)

nSamples = nrow(datExpr0)

plotTOM = dissTOM^7

diag(plotTOM) = NA

pdf(file="10_allgene_heatmap.pdf",width=9, height=9)

TOMplot(plotTOM, geneTree, moduleColors, main = "Network heatmap plot, all genes")

dev.off()

nSelect = 400

set.seed(10)

select = sample(nGenes, size = nSelect)

selectTOM = dissTOM[select, select]

selectTree = hclust(as.dist(selectTOM), method = "average")

selectColors = moduleColors[select]

plotDiss = selectTOM^7

diag(plotDiss) = NA

pdf(file="11_selectgene_heatmap.pdf",width=9, height=9)

TOMplot(plotDiss, selectTree, selectColors, main = "Network heatmap plot, selected genes")

dev.off()

pdf(file="12_module_dendrogram.pdf",width=6, height=6)

par(cex = 1.0)

plotEigengeneNetworks(MEs, "Eigengene dendrogram", marDendro = c(0,4,2,0), plotHeatmaps = FALSE)

dev.off()

pdf(file="13_module_heatmap.pdf",width=6, height=6)

par(cex = 1.0)

plotEigengeneNetworks(MEs, "Eigengene adjacency heatmap", marHeatmap = c(3,4,2,2), plotDendrograms = FALSE, xLabelsAngle = 90)

dev.off()

pdf(file="14_dendrogram_heatmap.pdf", width=5, height=7.5)

par(cex = 0.9)

plotEigengeneNetworks(MEs, "", marDendro = c(0,4,1,2), marHeatmap = c(3,4,1,2), cex.lab = 0.8, xLabelsAngle= 90)

dev.off()

cytoDir="CytoscapeInput"

dir.create(cytoDir)

for (mod in 1:nrow(table(moduleColors)))

{

modules = names(table(moduleColors))[mod]

probes = names(datExpr0)

inModule = (moduleColors == modules)

modProbes = probes[inModule]

modGenes = modProbes

modTOM = TOM[inModule, inModule]

dimnames(modTOM) = list(modProbes, modProbes)

edges_File = paste("CytoscapeInput-edges-", modules , ".txt", sep="")

nodes_File = paste("CytoscapeInput-nodes-", modules, ".txt", sep="")

outEdge=paste(cytoDir,edges_File,sep="\\")

outNode=paste(cytoDir,nodes_File,sep="\\")

cyt = exportNetworkToCytoscape(modTOM,

edgeFile = outEdge,

nodeFile = outNode,

weighted = TRUE,

threshold = 0.02,

nodeNames = modProbes,

altNodeNames = modGenes,

nodeAttr = moduleColors[inModule])

}

Note: After running the 90st line of code, we removed outliers by hand.

The table named “normalize.txt” contains the gene expression data downloaded from GEO database. The data was normalized. The table named “input.txt” contains the expression data of top 5000 genes. The table named “type.txt” contains the clinical data of the samples.
